# Two-way associations between relationship quality and uptake of couples health screening including HIV testing and counselling together: quantitative analysis of a couples cohort in rural South Africa

**DOI:** 10.1080/09540121.2024.2308741

**Published:** 2024-02-21

**Authors:** Fatma Abdelkhalek, Phillip Joseph, Laurie DeRose, Emmanuel Olamijuwon, Pumla Dladla, Thulani Ngubane, Victoria Hosegood, Heidi van Rooyen, Alastair van Heerden, Nuala McGrath

**Affiliations:** a CHERISH programme, School of Primary Care, Population Sciences and Medical Education, Faculty of Medicine, University of Southampton, Southampton, UK; b Faculty of Commerce, Assiut University, Assiut, Egypt; c Human Sciences Research Council, Sweetwaters, Pietermaritzburg, South Africa; d Catholic University of America, Washington DC, USA; e School of Geography and Sustainable Development, University of St. Andrews, UK; f Department of Social Statistics & Demography, Faculty of Social Sciences, University of Southampton, Southampton, UK; g SAMRC-WITS Developmental Pathways for Health Research Unit, Faculty of Health Sciences, University of the Witwatersrand, Johannesburg, South Africa; h Africa Health Research Institute, KwaZulu-Natal, South Africa

**Keywords:** Couples, HIV testing and counselling, relationship quality, behavioural intervention, South Africa, SDG 3: Good health and well-being

## Abstract

In the context of a couples cohort established to evaluate an optimised couples-focused behavioural intervention in rural South Africa, we examined: (1) Is couples’ relationship quality (RQ) associated with couples HIV testing and counselling (CHTC) uptake? (2) Does CHTC uptake or the intervention components uptake improve subsequent RQ? Enrolled couples, (*n* = 218), previously naïve to couples HIV testing, were invited to two group sessions and offered four couples counselling sessions (CS1-CS4), as part of the intervention and administered a questionnaire individually at baseline, four weeks, and four months, which included item-scales to measure RQ: satisfaction, intimacy, dyadic trust, conflict, and mutual constructive communication. Logistic models indicated that no baseline RQ measures were significantly associated with CHTC uptake. Linear regression models showed that CHTC uptake before four weeks assessment significantly improved couples’ satisfaction and trust at four weeks, and intimacy at four months. Attending at least one CS was associated with increased satisfaction, intimacy, and decreased conflict within couples at four weeks; the improvement in intimacy was sustained at four months. Consistent with the theoretical interdependence model, our findings suggest that CHTC and CS seemed to strengthen aspects of relationship quality, possibly leading to further collaboration in managing lifestyle changes and treatment adherence.

## Introduction

Theoretically, the interdependence model supports that couples who are motivated by a relationship-centered outlook are more likely to act jointly in initiating and sustaining health behaviour change and promoting treatment adherence (Lewis et al., 2006; Rogers et al., 2016). There is growing evidence that interventions targeting couples rather than individuals can achieve significantly greater improvements in behaviour change and in managing long-term health conditions including diabetes, cancer, HIV, or physical activities (Arden-Close & McGrath, 2017; Berry et al., 2017; Burton et al., 2010; Franks et al., 2018; Smith et al., 2023). In response to the HIV epidemic, couples-focused behavioural interventions have aimed to facilitate a transformation of motivation within the couple from an individual-level focus to caring for their health as a couple – thus becoming more willing to engage together to reduce sexual-risk behaviours and take action to achieve better health outcomes and improve relationship quality (Burton et al., 2010; Darbes et al., 2014). However, poor relationship quality may impede couples from achieving sexual risk behaviour change in the context of a targeted couples-based HIV prevention intervention (Ruark et al., 2018).

The World Health Organisation advocated Couples HIV Testing and Counselling (CHTC) for couples in or planning to be in a sexual relationship. In sub-Saharan Africa, CHTC is cost-effective relative to individual testing, in part because it is associated with greater engagement with HIV prevention and care, however, CHTC uptake has remained low (Jiwatram-Negrón & El-Bassel, 2014; Sibanda et al., 2017; Wall & Allen, 2017). To date, no quantitative research has investigated whether couples’ relationship quality influences CHTC uptake. Qualitative studies in African communities have shown that characteristics of low-quality relationships both impede and promote CHTC. Couples’ fear of HIV-positive results and the challenges of navigating post-CHTC relationships impede CHTC uptake, but mistrust and weak communication skills may motivate couples to seek information on joint sero-status through CHTC (Matovu et al., 2014; Nannozi, Wobudeya, & Gahagan, 2017; Nannozi, Wobudeya, Matsiko, et al., 2017). That is, the relational characteristics that are barriers to the private disclosure of HIV status to sexual partners could possibly motivate CHTC. The finding that good communication skills promote HIV disclosure of individual testing to sexual partners (Antelman et al., 2001; Kadowa & Nuwaha, 2009; Qiao et al., 2016) might suggest that couples with higher relationship quality should be more likely to test for HIV together (Nannozi et al., 2017b).

Undertaking CHTC can also be a strategy for building trust between couples (Matovu et al., 2014). However, CHTC has a varied impact on couples’ relationships depending on the HIV result status e.g., learning the sero-status together results in losing sexual intimacy, being concordant negatives enhances trust and fidelity among couples, while discordant status can be considered proof of infidelity (Tabana et al., 2013). Many couples remain together regardless of CHTC results, and stories about couples surviving testing can lend confidence to other wary couples (Tabana et al., 2013). Further, post-couples HIV testing counselling sessions may indirectly influence couples’ relationship quality e.g., increasing trust and relationship power and further promoting sexual risk reduction behaviours such as condom use (Allen et al., 2003; Bhushan et al., 2019).

Data from the Igugu Lethu “Our treasure” (IL) study, an optimised couples-focused behavioural intervention to promote CHTC uptake among heterosexual couples in rural KwaZulu-Natal, South Africa (McGrath et al., 2022), provides an opportunity to conduct a couple-level analysis that explores the two-way associations between five relationship quality measures and CHTC at two-time points (four weeks and four months). This secondary data analysis examines: (1) Is couples’ relationship quality at enrolment associated with CHTC uptake? (2) Does uptake of CHTC or uptake of the IL intervention components improve subsequent couples’ relationship quality?

## Method

### Study design

Igugu Lethu (IL) is a prospective cohort study. Participants were recruited using adverts of the IL study that were published and circulated on a large scale in the target community (McGrath et al., 2022). A short, initial screener was administered to determine the eligibility of individuals who showed interest in response to the study adverts. If the index individual was considered eligible, they were given an invitation to pass to their partner which asked the partner to contact the study team if interested. The initial screener was administered to interested partners and, if both partners were eligible, the couple was invited for baseline assessment. Eligible couples were aged 18+ years, had never previously tested together for HIV or mutually disclosed their HIV status, defined each other as a primary partner, had been together for at least six months, and neither partner reported intimate partner violence in the past six months. The study had two types of visits: assessment and intervention delivery visits. Each partner was administered a questionnaire individually at baseline, four weeks, and four months assessments. As part of the intervention, couples were invited to two group sessions [GS1 and GS2] and offered up to four couples counselling sessions [CS1-CS4]. Couples were offered CHTC as part of a broader health screening outcome, where health screening uptake without CHTC was not an option for couples. Health screening uptake was available for couples at any time after GS1. During health screening, couples were also offered self-sampling for curable STI testing, and measurement of blood pressure, random blood glucose, and BMI. Couples who took up health screening were further offered a post-health screening couples counselling session [post-HS CS] to further discuss their results and their plans for their future together. All counselling sessions were offered for each couple separately and led by a trained counsellor. Each enrolled couple was followed for four months. Detailed information about the IL study design has been published elsewhere (McGrath et al., 2022; Morton et al., 2021). The primary evaluation of the intervention compared this IL cohort to a historical cohort. The historical cohort did not have relationship quality measurements at four weeks or four months so the analysis for this paper is limited to the IL cohort.

### Measurement

### Relationship quality

Participants were asked to report their relationship quality at baseline, four weeks, and four months assessments using item scales that had been previously used in the same community (Darbes et al., 2014). Five relationship quality measures were constructed and used throughout the analysis. Each individual relationship quality measure is the aggregated sum of the responses for corresponding item scales for each participant at each time point. Details of these scales are in Appendix A. In brief:

- Satisfaction was measured by a single item. Responses ranged from 1 (not at all satisfied) to 6 (completely satisfied).
- Intimacy was measured using a six-item intimacy subscale (Kurdek, 1996). Response for each item ranged from 1 (not at all true) to 9 (very true); subscale total ranged 6–54.
- Trust was measured using an eight-item dyadic trust subscale (Larzelere & Huston, 1980). Response for each item ranged from 1 (strongly disagree) to 7 (strongly agree); subscale total ranged 8–56.
- Mutual constructive communication was measured by a three-item subscale (Christensen & Shenk, 1991). Response for each item ranged from 1 (very unlikely) to 9 (very likely): subscale total ranged 3–27
- Conflict within the couple was measured by a single item, with responses coded from 1 (often) to 3 (rarely).

For consistency, negative items within each measure were reversed e.g., the responses to the item “I feel that my partner does not show me enough consideration” within the trust scale, were reversed such that 1 (Strongly agree) to 7 (Strongly disagree). This way, for all relationship measures the higher the score, the better the relationship. The couple-level relationship quality measures used in the analysis are (a) the average of each scale between the two partners, and (b) the difference obtained by subtracting the female partner’s score from the male partner’s score.

### Couples HIV testing and counselling

Two binary indicators were created to represent whether couples participated in the health screening including CHTC offered by the IL study by four weeks and by four months.

### Intervention components and post-health screening couple counselling session

All enrolled couples had attended GS1. Therefore, the intervention components were represented in models by (1) a binary indicator for the attendance of GS2, and (2) another binary indicator of whether couples had attended at least one couple counselling session (pre-CHTC). We also included a binary indicator for whether the couple had attended the counselling session offered after their health screening (post-HS CS, not part of the intervention) as this could affect relationship quality independently of CHTC and intervention components uptake. 80% of couples who attended the post-HS CS had done so after the four weeks assessment visit, hence, the post-HS CS was included only in the four months analysis.

### Other variables

At baseline, participants were asked about their background characteristics (age, highest educational attainment, and employment status). They were also asked about their HIV testing history. For our analysis, we created couple-level variables for all characteristics. Couple age was dichotomised using the traditional African aging definition (Kowal & Dowd, 2001) to differentiate between younger and older couples (0 = Couples less than 50 years; 1 = Couples with at least one partner aged 50+ years). Other characteristics were represented by categorical variables: couple education (1 = Both less than grade 12; 2 = Both grade 12+; 3 = Male-only less than grade 12; 4 = Female-only less than grade 12), couple employment (1 = Both employed; 2 = Neither employed; 3 = Male-only employed; 4 = Female-only employed), and couple HIV testing history (1 = Both ever tested; 2 = Neither ever tested; 3 = Male-only tested; 4 = Female-only tested). We considered these variables as potential confounders of the two-way associations between relationship quality and CHTC uptake based on previous literature (Afari et al., 2022; Alimoradi et al., 2022; Fan et al., 2018). Other couple characteristics were also captured at baseline assessment, including relationship duration, marital status and cohabitation status. We chose not to control for variables like relationship duration, shared pregnancy, and cohabitation status which can be a result of couples relationship quality: when controlled, the total effect of relationship quality on the odds of CHTC uptake would likely be underestimated.

### Statistical analysis

Descriptive statistics were calculated using Chi-squared test for categorical variables and Wilcoxon rank sum test or Kruskal–Wallis test for continuous variables to explore how couples’ background characteristics, as well as relationship quality measures at baseline, were associated with CHTC uptake and subsequent relationship quality.

For research question 1, we used logistic regression to explore the odds of CHTC uptake by four weeks as a function of couple-level (average and difference) relationship quality measures at baseline after adjustment for potential confounders. Using the same approach, we modelled the odds of CHTC uptake by four months.

For research question 2, we used linear regression models for the average of each couple’s relationship quality measure at four weeks and again at four months. To isolate the effect of CHTC, we controlled for GS2 attendance, attending at least one couple CS, the post-HS CS attendance, and couple-level (average and difference) relationship quality at baseline, and considered possible confounders.

In the four weeks analysis, the intervention components were represented as two dichotomous variables: attending GS2 (0 = No; 1 = Yes) and whether each couple had attended at least one couple CS before four weeks assessment visit (0 = No couple CS; 1 = Attended at least one couple CS, only two couples had completed two couple CS before their four weeks assessment). In the four months analysis, there was no difference in the effect size between different number of couple CS attended, so the couple CS component of the intervention was again represented by a dichotomous indicator (0 = No couple CS; 1 = Attended at least one couple CS). The health screening including CHTC uptake was represented by a categorial variable (1 = Never screened; 2 = Screened before four weeks assessment; 3 = Screened after four weeks assessment).

For both research questions, we first conducted univariate regression and used the likelihood ratio test (LRT) to identify variables to be considered factors in multivariable analysis. For the final multivariable models, we again used the LRT to assess the contribution of each variable, and only significant confounders at the 5% level were retained in the final multivariable models.

We conducted two sensitivity analyses. For research question 1, to explore whether our results were different among those with no history within their relationship of an HIV positive result from previous testing, we re-ran the final models excluding couples who reported at their baseline assessment at least one HIV-positive result from previous testing. Thus, this sensitivity analysis included couples who had previously never tested for HIV and those who had at least one partner that had previously tested and received an HIV negative result. For research question 2, to examine the impact of any HIV-positive result at CHTC on couples subsequent relationship quality, we replaced our previous indicator of CHTC uptake before four weeks with a categorical variable distinguishing the different possible combinations of HIV results at CHTC compared to no CHTC (1 = Never/Screened after four weeks; 2 = Two HIV-negative results; 3 = One HIV-positive result; 4 = Two HIV-positive results). In the four months analysis, we simplified the previous three level category of CHTC uptake timing (before 4 weeks, after 4 weeks vs never) and used the same new categorical variable, distinguishing the different combination of HIV results possible from CHTC compared to no CHTC, used in the four weeks analysis. All analyses were performed using Stata 17.

## Results

Five hundred and sixty-nine (569) index individuals were screened and 398 partners. Three hundred and ninety-three (393) couples were invited for baseline assessment, with 86% completion. Three hundred and twenty-nine (329) eligible couples were invited to a first group session (GS1) and 223 (68%) attended and thus enrolled. Five couples were retrospectively excluded from the study based on information that came to light during their engagement with the study team after GS1, resulting in 218 eligible couples enrolled in the IL study.

## Descriptive statistics

Table 1 describes participation in the intervention components, assessment visits, and uptake of CHTC among the IL enrolled couples (*n* = 218). Two hundred and ten (96%) reported that they were not married. The retention over 4 months follow-up and participation in follow-up assessments was high (> = 89%). As part of the intervention, 167 (77%) attended GS2, and 113 (52%) attended at least one couple CS. A total of 122 (56%) took up CHTC by four months. Couples who took up CHTC took up all the other health tests offered, 77 (35%) of them chose to do so before four weeks, and 100 (82%) attended a post-HS CS.

**Table 1.** Participation in components of the Igugu Lethu study by enrolled couples.

IL Study	*n* (%)
***Marital status***	
Married	8 (4)
Not married	210 (96)
***Intervention components***	
**GS1**	
Yes	218 (100)
**GS2**	
Yes	167 (76.6)
No	51 (23.4)
**At least one couple CS**	
Yes	113 (51.8)
No	115 (48.2)
**Health screening, including CHTC**	
Screened before four weeks assessment	77 (35.3)
Screened after four weeks assessment	45 (20.6)
Never screened	96 (44.0)
**Post-HS CS**	
Yes	100 (81.9)
No	22 (18.1)
**Assessment *visits***	
Baseline	218 (100)
Four weeks	208 (95.4)
Four months	195 (89.5)
**n**^£^	218

Note: ^£^Total number of enrolled couples in the IL study.

Table 2 shows the descriptive statistics of couple-level relationship quality as well as couples’ characteristics at baseline according to CHTC uptake while Table 3 presents the variability of couple-level average of each subsequent relationship quality measure by couples’ characteristics at baseline. The results indicate that couple-level average of each relationship quality measure at baseline was close to the upper limit of each scale, and none were significantly associated with CHTC uptake status by four weeks or four months (Table 2). Among couples’ characteristics, only couple age was significantly associated with CHTC uptake by four weeks (*p* = 0.002), and by four months (*p* = 0.02), where older couples with at least one partner aged 50+ years were more likely to take up CHTC compared to younger couples (Table 2). Similarly, older couples had significantly higher intimacy and trust scores compared to younger couple and were more satisfied with their relationship (four months only) (Table 3).

**Table 2.** Couple-level relationship quality measures and couples’ characteristics at baseline by CHTC uptake status by four weeks and by four months.

	CHTC uptake by four weeks			CHTC uptake by four months		
	Yes, by four weeks	Never / yes, after four weeks	*p*-Value	Yes	No	*p*-Value
**Couple-level relationship quality at baseline**						
	Median (IQR)^a^			Median (IQR) ^a^		
Average- satisfaction	6 (5.5, 6)	6 (5.5, 6)	.384	6 (5.5, 6)	6 (5.5, 6)	.563
Difference- satisfaction	0 (0, 1)	0 (0, 0)	.502	0 (0, 1)	0 (0, 0)	.686
Average- trust	49.5 (45.5, 53)	48.5 (44, 52.5)	.325	49 (45, 52.5)	48.8 (44, 53)	.840
Difference- trust	3 (−3, 8)	3 (−1, 11)	.269	4 (−2, 9)	.5 (−2.5, 11)	.512
Average- intimacy	48 (44.5, 52)	47.5 (44, 52)	.227	47 (43.5, 52)	48.3 (44.3, 52)	.416
Difference- intimacy	2 (0,12)	4 (0, 12)	.565	4 (0, 14)	4 (0, 9)	.248
Average- communication	25.5 (24.5, 27)	25.5 (23, 27)	.114	25.5 (23, 27)	25.5 (23, 27)	.802
Difference- communication	0 (0, 3)	3 (−3, 8)	.256	0 (0, 3)	0 (0, 3)	.719
Average- conflict	2.5 (2.5, 3)	2.5 (2.5, 3)	.546	2.5 (2.5, 3)	2.5 (2.5, 3)	.491
Difference- conflict	0 (0, 1)	0 (0, 1)	.747	0 (0, 1)	1 (0, 1)	.413
	*N* (%)^b^			*N* (%)^b^		
**Couple age**						
≤50 years	73 (78.5)	116 (92.8)	.002**	100 (81.9)	89 (92.7)	.02*
51+ years	20 (21.5)	9 (7.2)		22 (18.3)	7 (7.3)	
**Couple education^£^**						
Both grade 12+	30 (32.6)	41 (33.1)	.349	38 (31.4)	33 (34.7)	.374
Both less than grade 12	33 (35.9)	32 (25.8)		42 (34.7)	23 (24.2)	
Male-only less than grade 12	11 (12.0)	22 (17.7)		18 (14.9)	15 (15.8)	
Female-only less than grad	18 (19.6)	29 (23.4)		23 (19.0)	24 (25.3)	
**Couple employment**						
Both employed	7 (7.5)	11 (8)	.931	9 (7.4)	9 (9.4)	.887
Neither employed	54 (58.1)	68 (54.4)		69 (56.6)	53 (55.2)	
Male-only employed	18 (19.4)	24 (19.2)		25 (20.5)	17 (17.7)	
Female-only employed	14 (15.1)	22 (17.6)		19 (15.57)	17 (17.7)	
**Couple (HIV) testing history^±^**						
Both ever tested	66 (71.7)	93 (75)	.506	87 (71.9)	72 (75.8)	.884
Neither ever tested	4 (4.3)	9 (7.6)		7 (5.8)	6 (6.3)	
Male-only tested	6 (6.5)	4 (3.2)		6 (4.9)	4 (4.2)	
Female-only tested	16 (17.4)	18 (14.5)		21 (17.4)	13 (13.9)	
n	93	125		122	96	

Note: Significant level: *<.05; **<.01. Test statistic: ^a^Exact-Wilcoxon rank sum test; ^b^Chi-square test. ^±^Two couples had incomplete data on HIV testing history because one partner did not report whether they had tested for HIV in the past. ^£^Two couples had incomplete data regarding the highest education level attained.

**Table 3.** Descriptive statistics of subsequent relationship quality measures at four weeks and at four months by couples’ characteristics at baseline.

	Average relationship quality at four weeks					Average relationship quality at four Months				
	Satisfaction	Trust	Intimacy	Communications	Conflict	Satisfaction	Trust	Intimacy	Communications	Conflict
	Median (IQR)					Median (IQR)				
Couples’ characteristics at baseline										
***Couple age***^a^										
≤ 50 years	6 (5.5, 6)	48 (44, 51.5)	46.5 (42.5, 50)	25.5 (23.7, 27)	2.5 (2.5, 3)	5.5 (5, 6)	45.5 (41, 49.5)	44 (40, 48)	24.5 (22.5, 26.5)	2.5 (2.5, 3)
51+ years	6 (5.75, 6)	52.7 (49, 54.3)	49.7 (47.5, 52.7)	26.7 (25.2, 27)	2.5 (2.2, 2.5)	6 (5.5, 6)	51.5 (47, 55.5)	50 (47.5, 52)	25 (23, 27)	2.5 (2, 3)
*p*-value	.08	.002**	.0002**	.08	.52	.01*	.0001**	.0001**	.14	.34
***Couple education***^b,£^										
Both grade 12+	6 (5.5, 6)	46.5 (43.5, 51)	46 (43, 49.5)	25 (24, 27)	2.5 (2.5, 3)	5.5 (5, 6)	44 (41, 49)	44 (39, 48.5)	24 (22, 27)	2.5 (2.5, 3)
Both less than grade 12	6 (6, 6)	50 (45.7, 53)	49.5 (43, 52.5)	26.5 (25, 27)	2.5 (2.5, 2.5)	5.5 (5.5, 6)	47.5 (41.5, 52.5)	46 (40, 50)	25.5 (23, 27)	2.5 (2.5, 3)
Male- only less than grade 12	6 (5.5, 6)	48 (42.7, 52.2)	47 (42.7, 50.5)	25.5 (22.7, 27)	2.5 (2.5, 3)	5.5 (5, 6)	46 (41.5, 48.5)	45.2 (38, 49.5)	24.2 (21, 25.5)	2.5 (2.5, 3)
Female- only less than grade 12	5.5 (5.5, 6)	48.5 (46, 51.5)	47 (42.5, 50)	25.5 (23, 27)	2.5 (2.5, 2.5)	5.5 (5, 6)	46.5 (42, 51.5)	46 (42.5, 47.5)	23.5 (22.5, 26)	2.5 (2.5, 3)
*p*-value	.05	.05	.054	.34	.71	.60	.27	.46	.02*	.39
***Couple employment***^b^										
Both employed	6 (5.5, 6)	47 (43.7, 52)	46.3 (42.7, 51)	25.5 (22.7, 27)	2.5 (2, 2.5)	5.5 (4.7, 6)	46.2 (38.2, 51.7)	44.5 (37, 49.3)	24.2 (20.2, 27)	2.5 (2.5, 2.5)
Neither employed	6 (5.5, 6)	48.7 (45, 52)	47 (43, 50)	26 (25, 27)	2.5 (2.5, 3)	5.5 (5, 6)	46.5 (42, 50.7)	44.5 (40.2, 48)	24.2 (22.5, 26.5)	2.5 (2.5, 3)
Male-only employed	6 (5.5, 6)	47.5 (44.5, 51.5)	46.5 (44, 50)	26 (23, 27)	2.5 (2, 2.5)	5.5 (5, 6)	43.5 (39.5, 50)	46 (39, 50)	25 (22.5, 26.5)	2.5 (2.5, 3)
Female-only employed	6 (5.5, 6)	51 (45, 53)	49.5 (44.5, 51)	25.5 (23.5, 26.5)	2.5 (2.5, 3)	5.5 (5.5, 6)	47 (39.5, 52.5)	44 (41.5, 49.5)	24.5 (22.5, 27)	2.5 (2.5, 2.5)
*p*-value	.47	.57	.88	.47	.12	.36	.68	.90	.95	.07
***Couple (HIV) testing****history^b,^*^£^										
Both ever tested	6 (5.5, 6)	45 (44, 52)	46.5 (42.5, 50)	25.5 (23, 27)	2.5 (2.3, 3)	5.5 (5, 6)	44.5 (41, 50.3)	44 (39.5, 48)	24.3 (22, 27)	2.5 (2.5, 3)
Neither ever tested	6 (5.5, 6)	52 (46.5, 53)	51.5 (40.5, 52.5)	27 (27, 27)	2.5 (2.5, 2.5)	6 (5.5, 6)	47.5 (39.5, 53)	44.5 (40, 52)	23 (23, 25)	2.5 (2.5, 3)
Male-only tested	6 (6, 6)	49.5 (47, 51.5)	47 (45, 52)	26 (25.5, 27)	2(2, 2.5)	6 (5.5, 6)	48.5 (42, 50)	46.5 (44, 50)	25 (22.5, 26.5)	2.5 (2.5, 3)
Female-only tested	6 (5.5, 6)	49.5 (45.5, 52)	47 (45.5, 50)	25.5 (23.5, 27)	2.5 (2.5, 3)	5.5 (5, 6)	47.5 (45.7, 41)	46 (43.3, 48.5)	25.3 (23, 27)	2.5 (2.5, 3)
*P*-value	0.40	0.19	0.49	0.04*	0.21	0.48	0.27	0.28	0.30	0.66
n	208^c^	208^c^	208^c^	208^c^	208^c^	195^d^	195^d^	195^d^	195^d^	195^d^

Note: Significant level: *<.05; **<.01. Test statistic: ^a^Exact-Wilcoxon rank sum test; ^b^Kruskal-Wallis test. ^±^Two couples had incomplete data on HIV testing history because one partner did not report whether they had tested for HIV in the past. ^£^Two couples had incomplete data regarding the highest education level attained. ^c^Total number of couples who completed 4 weeks assessment; ^d^Total number of couples who completed 4 months assessment.

## Relationship quality and couples HIV testing and counselling uptake

The multivariable model results showed that none of the couple-level relationship quality measures at baseline were significantly associated with CHTC uptake either by four weeks (Table 4) or four months (Table 5), after adjustment for couples’ characteristics at baseline. In our sensitivity analysis, 61 couples (29% of enrolled couples) were dropped, of which 21 (34%) were concordant HIV positive. A comparison of the sensitivity analysis results with estimates in Tables 4 and 5 determined that the size of the adjusted ORs and the 95%CI estimates for each relationship quality measure remained virtually unchanged, data not shown.

**Table 4.** Final muiltivariable logistic regressions examining the association between relationship quality measures at baseline and the odds of CHTC uptake by four weeks.

	(1)	(2)	(3)	(4)	(5)
	CHTC-4w OR (95% CI)	CHTC-4w OR (95% CI)	CHTC-4w OR (95% CI)	CHTC-4w OR (95% CI)	CHTC-4w OR (95% CI)
***Couple-level relationship quality at baseline***					
Average- satisfaction	1.88				
	(0.89–4.01)				
Difference- satisfaction	1.27				
	(0.84–1.92)				
Average- trust		1.00			
		(0.95–1.06)			
Difference- trust		0.98			
		(0.95–1.01)			
Average- intimacy			1.03		
			(0.97–1.10)		
Difference- intimacy			1.00		
			(0.97–1.04)		
Average- communication				1.06	
				(0.95–1.19)	
Difference- communication				0.97	
				(0.91–1.03)	
Average- conflict					0.70
					(0.31–1.61)
Difference- conflict					0.87
					(0.59–1.30)
***Couple age***					
≤ 50 years	reference	reference	reference	reference	reference
51+ years	3.64**	3.81**	3.54**	3.54**	3.66**
	(1.44–9.19)	(1.48–9.78)	(1.40–8.95)	(1.40–8.96)	(1.46–9.21)
***Couple education***					
Both grade 12+	reference	reference	reference	reference	reference
Both less than grade 12	0.92	1.01	0.97	1.00	1.02
	(0.43–1.97)	(0.47–2.17)	(0.45–2.09)	(0.47–2.15)	(0.48–2.19)
Male- only less than grade 12	0.68	0.71	0.70	0.74	0.72
	(0.27–1.69)	(0.29–1.76)	(0.28–1.73)	(0.30–1.84)	(0.29–1.78)
Female-only less than grade 12	0.74	0.79	0.76	0.73	0.81
	(0.33–1.65)	(0.35–1.77)	(0.34–1.68)	(0.33–1.62)	(0.37–1.81)
***Couple employment***					
Both employed	0.70	0.69	0.68	0.73	0.60
	(0.24–2.08)	(0.23–2.07)	(0.23–1.98)	(0.24–2.17)	(0.19–1.86)
Neither employed	reference	reference	reference	reference	reference
Male-only employed	0.97	1.01	0.99	1.09	1.01
	(0.46–2.04)	(0.48–2.12)	(0.47–2.08)	(0.51–2.32)	(0.48–2.12)
Female-only employed	0.80	0.74	0.77	0.77	0.77
	(0.35–1.83)	(0.33–1.66)	(0.34–1.76)	(0.34–1.72)	(0.34–1.74)
***Couple (HIV) testing history***					
Both never tested	reference	reference	reference	reference	reference
Neither ever tested	0.48	0.50	0.51	0.53	0.52
	(0.13–1.76)	(0.14–1.85)	(0.14–1.88)	(0.14–2.00)	(0.14–1.95)
Male-only tested	2.29	1.95	2.04	1.98	2.11
	(0.56–9.40)	(0.50–7.59)	(0.53–7.91)	(0.52–7.56)	(0.55–8.09)
Female-only tested	1.25	1.30	1.39	1.35	1.35
	(0.56–2.80)	(0.58–2.90)	(0.62–3.11)	(0.60–3.04)	(0.60–3.02)
n^£^	213	213	213	213	213

Note: Significant level: *<.05; **<.01. ^£^The models exclude: two couples had incomplete data on HIV testing history because one partner did not report whether they had tested for HIV in the past, another couple was excluded where the female partner reported a disclosure of her positive HIV test result to her partner, and another two couples had incomplete data regarding the highest education level attained.

**Table 5.** Final multivariable logistic regressions examining the association between relationship quality measures at baseline and the odds of CHTC uptake by four months.

	(1)	(2)	(3)	(4)	(5)
	CHTC-4m OR (95% CI)	CHTC-4m OR (95% CI)	CHTC-4m OR (95% CI)	CHTC-4m OR (95% CI)	CHTC-4m OR (95% CI)
***Couple-level relationship quality at baseline***					
Average- satisfaction	1.35				
	(0.76–2.38)				
Difference- satisfaction	1.05				
	(0.77–1.43)				
Average- trust		1.00			
		(0.95–1.05)			
Difference- trust		1.00			
		(0.98–1.03)			
Average- intimacy			0.99		
			(0.93–1.05)		
Difference- intimacy			1.02		
			(0.99–1.06)		
Average- communication				0.98	
				(0.88–1.09)	
Difference- communication				0.98	
				(0.92–1.04)	
Average- conflict					0.63
					(0.27–1.46)
Difference- conflict					0.79
					(0.52–1.19)
***Couple age***					
≤ 50 years	reference	reference	reference	reference	reference
51+ years	2.63	2.68*	2.84*	2.77*	2.65*
	(1.00–6.93)	(1.00–7.14)	(1.07–7.51)	(1.05–7.31)	(1.01–6.98)
***Couple Education***					
Both grade 12+	reference	reference	reference	reference	reference
Both less than grade 12	1.15	1.21	1.24	1.22	1.23
	(0.54–2.45)	(0.57–2.57)	(0.58–2.66)	(0.58–2.59)	(0.58–2.61)
Male-only less than grade 12	0.98	0.99	0.91	0.99	1.05
	(0.41–2.32)	(0.42–2.34)	(0.38–2.18)	(0.42–2.36)	(0.44–2.50)
Female-only less than grade 12	0.76	0.75	0.78	0.75	0.83
	(0.35–1.63)	(0.35–1.63)	(0.36–1.68)	(0.35–1.62)	(0.38–1.80)
***Couple employment***					
Both employed	0.68	0.70	0.75	0.67	0.56
	(0.24–1.94)	(0.25–1.99)	(0.26–2.15)	(0.24–1.91)	(0.19–1.68)
Neither employed	reference	reference	reference	reference	reference
Male-only employed	1.15	1.18	1.23	1.22	1.17
	(0.55–2.39)	(0.57–2.45)	(0.59–2.56)	(0.58–2.55)	(0.56–2.44)
Female-only employed	0.81	0.84	0.92	0.82	0.80
	(0.37–1.80)	(0.38–1.84)	(0.41–2.02)	(0.37–1.78)	(0.36–1.75)
***Couple (HIV) testing history***					
Both ever tested	reference	reference	reference	reference	reference
Neither ever tested	0.75	0.81	0.78	0.84	0.83
	(0.23–2.46)	(0.24–2.66)	(0.24–2.59)	(0.25–2.76)	(0.25–2.76)
Male-only tested	1.16	1.15	1.21	1.11	1.20
	(0.30–4.54)	(0.30–4.41)	(0.32–4.64)	(0.29–4.26)	(0.31–4.61)
Female-only tested	1.34	1.39	1.34	1.39	1.40
	(0.59–3.03)	(0.62–3.12)	(0.59–3.03)	(0.62–3.13)	(0.62–3.15)
n^£^	213	213	213	213	213

Note: Significant level: *<.05; **<.01. ^£^The models exclude: two couples had incomplete data on HIV testing history because one partner did not report whether they had tested for HIV in the past, another couple was excluded where the female partner reported a disclosure of her positive HIV test result to her partner, and another two couples had incomplete data regarding the highest education level attained.

## Couples HIV testing and counselling, intervention components uptake and subsequent relationship quality

Turning to subsequent relationship quality, each of the five columns (models) in Table 6 corresponds to the average score of each relationship quality measure at four weeks (Table 6) and four months (Table 7), which is interpreted as change in relationship quality measure by 4 weeks as baseline relationship quality score is included in the models. CHTC uptake before four weeks significantly improved couples’ satisfaction (0.21, 95% CI (0.07, 0.36)-Model 1), and couples’ trust (1.99, 95% CI (0.55, 3.44)-Model 2) at 4 weeks compared to those who had not taken up CHTC, after adjustment for the intervention components, couples HIV testing history, and the corresponding couple-level relationship quality measure at baseline (Table 6). In contrast, CHTC uptake or its timing had no significant association with relationship quality measures at four months, except for intimacy. The intimacy score between couples significantly increased from baseline by about 4 points, on average, at four months among couples who took up CHTC (irrespective of the timing) compared to those who had not taken up CHTC, after adjustment for the intervention components, post-HS CS, couple age, couple HIV testing history (Model 3, Table 7). Table 6 also reveals the effect of the intervention components on subsequent relationship quality. Attending at least one couple CS before four weeks assessment, as part of the intervention, significantly increased couples’ satisfaction (0.17, 95% CI (0.00, 0.33)-Model 1) and intimacy (1.97, 95% CI (0.22, 3.72)-Model 3), as well as decreased conflict within couples (−0.22, 95% CI (−0.35, −0.10)-Model 5) at four weeks compared to not attending a couple CS, holding all else constant (Table 6). The observed effect of the intervention on improving couples’ relationship quality at four weeks was sustained only for couples’ intimacy at four months. Attending at least one couple CS significantly improved couples’ intimacy (2.20, 95% CI (0.35, 4.04)-Model 3) (Table 7).

**Table 6.** Final multivariable linear regressions examining the association between the intervention components, CHTC uptake and each relationship quality measure at four weeks.

	(1)	(2)	(3)	(4)	(5)
	Satisfaction *β* (95% CI)	Trust *β* (95% CI)	Intimacy *β* (95% CI)	Communication *β* (95% CI)	Conflict *β* (95% CI)
***Intervention components***					
***GS2***					
No	reference	Reference	reference	reference	reference
Yes	0.05	−0.05	0.36	0.35	0.08
	(−0.11–0.21)	(−1.68–1.58)	(−1.33–2.05)	(−0.56–1.27)	(−0.05–0.20)
***At least one couple CS before 4 weeks visit***					
No	reference	Reference	reference	reference	reference
Yes	0.17*	0.84	1.97*	0.22	−0.22**
	(0.00–0.33)	(−0.82–2.50)	(0.22–3.72)	(−0.71–1.16)	(−0.35–−0.10)
***Health Screening, including CHTC***					
Never/Screened after 4 weeks visit	reference	Reference	reference	reference	reference
Screened before 4 weeks visit	0.21**	1.99**	1.51	0.60	0.01
	(0.07–0.36)	(0.55–3.44)	(−0.00–3.03)	(−0.22–1.42)	(−0.10–0.12)
***Couple (HIV) testing history^±^***					
Both ever tested	reference	Reference	reference	reference	reference
Neither ever tested	0.06	2.56	0.58	1.63*	0.07
	(−0.21–0.34)	(−0.20–5.33)	(−2.30–3.45)	(0.06–3.21)	(−0.14–0.28)
Male-only tested	0.20	0.76	0.77	1.18	−0.35**
	(−0.14–0.53)	(−2.55–4.06)	(−2.66–4.20)	(−0.69–3.04)	(−0.60–−0.10)
Female-only tested	0.11	2.37*	3.05**	−0.06	0.00
	(−0.08–0.30)	(0.46–4.27)	(1.07–5.03)	(−1.13–1.01)	(−0.14–0.14)
**Couple-level relationship quality at baseline**					
Average- satisfaction	0.25**				
	(0.12–0.38)				
Difference- satisfaction	0.03				
	(−0.05–0.10)				
Average- trust		0.50**			
		(0.39–0.62)			
Difference- trust		0.01			
		(−0.05–0.08)			
Average- intimacy			0.50**		
			(0.35–0.64)		
Difference- intimacy			−0.01		
			(−0.09–0.08)		
Average- communication				0.32**	
				(0.18–0.46)	
Difference- communication				0.03	
				(−0.04–0.11)	
Average- conflict					0.18*
					(0.03–0.33)
Difference- conflict					−0.02
					(−0.09–0.05)
*n*^£^	203	203	203	203	203

Note: Significant level: *<.05; **<.01. ^£^203 couples had completed relationship quality questions at baseline and four weeks assessments. The models exclude: two couples had incomplete data on HIV testing history because one partner did not report whether they had tested for HIV in the past, another couple was excluded where the female partner reported a disclosure of her positive HIV test result to her partner, and another two couples had incomplete regarding the highest education level attained.

**Table 7.** Final multivariable linear regressions examining the association between the intervention components, CHTC uptake and each relationship quality measure at four Months.

	(1)	(2)	(3)	(4)	(5)
	Satisfaction *β* (95% CI)	Trust *β* (95% CI)	Intimacy *β* (95% CI)	Communication *β* (95% CI)	Conflict *β* (95% CI)
***Intervention components***					
**GS2**					
No	reference	reference	reference	reference	reference
Yes	−0.14	0.35	−0.79	−0.64	0.10
	(−0.38–0.10)	(−1.78–2.48)	(−2.70–1.13)	(−1.63–0.35)	(−0.02–0.22)
***At least one couple CS***					
No	reference	reference	reference	reference	reference
Yes	0.19	1.14	2.20*	−0.22	0.06
	(−0.05–0.42)	(−0.94–3.21)	(0.35–4.04)	(−1.18–0.75)	(−0.06–0.18)
					
***Health Screening, including CHTC***					
Never	reference	reference	reference	reference	reference
Screened after 4 weeks visit	0.08	0.86	4.24**	−0.09	0.03
	(−0.27–0.43)	(−2.28–4.01)	(1.42–7.07)	(−1.56–1.38)	(−0.15–0.21)
Screened before 4 weeks visit	0.14	1.00	4.15*	−0.16	−0.13
	(−0.28–0.55)	(−2.67–4.67)	(0.88–7.43)	(−1.87–1.54)	(−0.34–0.08)
***Post-HS CS***					
No	reference	reference	reference	reference	reference
Yes	0.17	2.20	0.09	0.71	0.13
	(−0.18–0.52)	(−0.90–5.30)	(−2.67–2.84)	(−0.72–2.15)	(−0.05–0.30)
***Couples age***					
≤ 50 years	reference	reference	reference	reference	reference
51+ years	0.19	4.06**	4.20**	0.54	−0.06
	(−0.12–0.49)	(1.27–6.84)	(1.80–6.61)	(−0.71–1.80)	(−0.21–0.10)
***Couples (HIV) testing history***					
Both tested	reference	reference	reference	reference	reference
Neither tested	0.13	0.71	0.37	−0.36	0.07
	(−0.27–0.53)	(−2.84–4.27)	(−2.77–3.51)	(−2.01–1.29)	(−0.13–0.27)
Male-only tested	0.19	1.07	1.93	0.63	0.13
	(−0.29–0.67)	(−3.15–5.29)	(−1.82–5.68)	(−1.33–2.59)	(−0.11–0.37)
Female-only tested	0.21	2.65*	2.87*	1.07	−0.06
	(−0.07–0.49)	(0.18–5.13)	(0.68–5.07)	(−0.08–2.21)	(−0.20–0.08)
**Couple-level relationship Quality at baseline**					
Average- satisfaction	0.11				
	(−0.11–0.33)				
Difference- satisfaction	−0.17**				
	(−0.28–−0.05)				
Average- trust		0.22*			
		(0.05–0.39)			
Difference- trust		−0.02			
		(−0.12–0.07)			
Average- intimacy			0.24**		
			(0.07–0.41)		
Difference- intimacy			−0.09		
			(−0.19–0.01)		
Average- communication				0.10	
				(−0.06–0.26)	
Difference- communication				−0.05	
				(−0.13–0.04)	
Average- conflict					0.11
					(−0.04–0.26)
Difference- conflict					0.03
					(−0.04–0.10)
n^£^	191	191	191	191	191

Note: Significant level: *<.05; **<.01. ^£^191 couples had completed relationship quality questions at baseline and four months assessments. The models exclude: two couples had incomplete data on HIV testing history because one partner did not report whether they had tested for HIV in the past, another couple was excluded where the female partner reported a disclosure of her positive HIV test result to her partner, and another Two couples had incomplete data regarding the highest education level attained.

In Table 8, our new estimates for satisfaction at 4 weeks indicated no important differences in estimates between HIV results at CHTC compared to the overall estimate associated with CHTC uptake reported in Table 6. Table 8 also shows that couples who received two HIV negative results at CHTC were estimated to have no difference in their trust score at four weeks compared to couples who had not taken up CHTC, while couples with one HIV positive result had, on average, a 3-point higher trust score, and couples with two HIV positive results had almost a 2-point higher trust score, on average, compared to couples who had not taken up CHTC (only the former was statistically significant). This contrasts with the significant association in Table 6 between overall CHTC uptake and trust at four weeks. We also see in Table 8 suggestion of a divergence in the average intimacy score at four weeks estimates, with categories of at least one HIV positive result having a higher intimacy score improvement, on average, compared to couples who had two HIV negative results and couples who had not taken up CHTC although none of these estimates were statistically significant, consistent with our analysis in Table 6. In Table 7, CHTC uptake had no significant association with relationship quality measures at four months, except for intimacy. The intimacy score between couples significantly increased from baseline by about 4 points, on average, at four months among couples who took up CHTC (irrespective of the timing) compared to those who had not taken up CHTC. Table 9 provides evidence that intimacy score at four months was significantly higher for couples who took up CHTC, irrespective of the HIV results, compared to couples who had not taken up CHTC. The point estimates suggested a possibly greater increase in intimacy score at four months for couples with at least one HIV positive result compared to couples with HIV negative results, although the estimates were not statistically significant different, a pattern also seen at four weeks (Table 8).

**Table 8.** Final multivariable linear regressions examining the association between the intervention components, and HIV result at CHTC uptake before four weeks and each relationship quality measure at four weeks^#^.

	(1)	(2)	(3)	(4)	(5)
	Satisfaction *β* (95% CI)	Trust *β* (95% CI)	Intimacy *β* (95% CI)	Communication *β* (95% CI)	Conflict *β* (95% CI)
***Health Screening, including CHTC***					
Never/Screened after 4 weeks visit	reference	reference	reference	reference	Reference
Two HIV-negative results at CHTC (*n* = 26)	0.23*	1.00	1.16	0.68	0.10
	(0.02–0.44)	(−1.06–3.06)	(−1.01–3.32)	(−0.49–1.84)	(−0.06–0.26)
One HIV-positive result at CHTC (*n* = 22)	0.23*	3.22**	1.85	1.07	−0.05
	(0.01–0.45)	(1.02–5.41)	(−0.46–4.16)	(−0.17–2.31)	(−0.21–0.12)
Two HIV-positive results at CHTC (*n* = 18)	0.16	1.90	1.62	−0.08	−0.03
	(−0.08–0.40)	(−0.47–4.28)	(−0.89–4.12)	(−1.44–1.28)	(−0.21–0.15)
n^£^	203	203	203	203	203

^#^ The results are from models also adjusted for the intervention components, couples HIV testing history, and the corresponding couple-level relationship quality measure at baseline, data not shown. Significant level: *<.05; **<.01. ^£^203 couples had completed relationship quality questions at baseline and four weeks assessments. The models exclude: two couples had incomplete data on HIV testing history because one partner did not report whether they had tested for HIV in the past, another couple was excluded where the female partner reported a disclosure of her positive HIV test result to her partner, and another two couples had incomplete regarding the highest education level attained.

**Table 9.** Final multivariable linear regressions examining the association between the intervention components, HIV result at CHTC uptake at any time during the follow up and each relationship quality measure at four Months^#^.

	(1)	(2)	(3)	(4)	(5)
	Satisfaction *β* (95% CI)	Trust *β* (95% CI)	Intimacy *β* (95% CI)	Communication *β* (95% CI)	Conflict *β* (95% CI)
***Health Screening, including CHTC***					
Never	reference	Reference	reference	reference	reference
Two HIV-negative results at CHTC (*n* = 44)	0.08	−0.24	3.10*	−0.60	−0.03
	(−0.30–0.46)	(−3.61–3.13)	(0.11–6.10)	(−2.17–0.97)	(−0.23–0.16)
One HIV-positive result at CHTC (*n* = 39)	0.07	1.45	5.03**	0.58	−0.02
	(−0.32–0.47)	(−2.06–4.96)	(1.89–8.17)	(−1.05–2.20)	(−0.22–0.19)
Two HIV-positive results at CHTC (*n* = 31)	0.13	1.91	5.03**	−0.11	0.03
	(−0.27–0.53)	(−1.65–5.46)	(1.86–8.20)	(−1.75–1.54)	(−0.18–0.24)
n^£^	191	191	191	191	191

^#^ The results are from models also adjusted for the intervention components, post-HS CS, couple age, couple HIV testing history, data not shown. Significant level: *<.05; **<.01. ^£^191 couples had completed relationship quality questions at baseline and four months assessments. The models exclude: two couples had incomplete data on HIV testing history because one partner did not report whether they had tested for HIV in the past, another couple was excluded where the female partner reported a disclosure of her positive HIV test result to her partner, and another Two couples had incomplete data regarding the highest education level attained.

## Discussion

This quantitative study is the first to examine the two-way associations between five couples’ relationship quality measures and the uptake of couples HIV testing and counselling (CHTC), in the context of a couples cohort conducted to evaluate a couples-focused behavioural intervention to promote CHTC.

The existing qualitative literature on barriers to CHTC uptake (Matovu et al., 2014; Nannozi, Wobudeya, & Gahagan, 2017; Nannozi, Wobudeya, Matsiko, et al., 2017) and disclosure of HIV test results to a sexual partner (Antelman et al., 2001; Kadowa & Nuwaha, 2009; Qiao et al., 2016) suggest both positive and negative effects of relationship quality on uptake of CHTC.

Our quantitative analysis found that baseline relationship quality measures were not significantly associated with the subsequent uptake of CHTC. While this is partially consistent with the qualitative literature, the lack of relationship could derive from significant aspects of relationship quality not being captured by our five constructed relationship quality measures or due to couples with relatively high relationship quality agreeing to participate in the IL study (selection bias). Couples with poor relationships may have resisted participating together in the study and all relationship quality measures at baseline were concentrated at the high-end of each measure spectrum. Further, almost 3 in 4 couples in this cohort had previously tested for HIV, which is to be expected within the context of wide availability of HIV treatment in South Africa, but many HIV negative results were from tests conducted some time ago and couples were only eligible for this study if they had never mutually disclosed an HIV test result to each other. The barriers to such disclosure did not seem to be reflected in their high scoring at baseline on our relationship quality measures.

On the other hand, the results are in accordance with the “transformation of motivations” concept described in the interdependence theory (Lewis et al., 2006) and supported by couples-focused behavioural interventions that promote sexual-risk behaviour change and influence couples’ emotional well-being (Burton et al., 2010; Darbes et al., 2014). We found that attending at least one couple counselling session as part of the intervention components was associated with increased satisfaction, intimacy, and decreased conflict within couples at four weeks, and the improvement in intimacy was sustained at four months. While couples who participated in CHTC experienced improvements in satisfaction and trust at four weeks as well as intimacy at four months, compared to those who did not undergo CHTC. The improvement in trust between couples is consistent with (Matovu et al., 2014) who showed that CHTC can be a way to build trust. On the contrary to previous findings (Tabana et al., 2013), our results showed also improvement in couples relationship quality (specifically trust and intimacy) among couples who had at least one HIV positive result at CHTC, compared to those who had not taken up CHTC.

In the context of Sustainable Development Goal 3 (SDG 3), which aims to ensure healthy lives and promote well-being for all at all ages, offering a broader health screening to couples ready to take up CHTC, contributes to several specific targets within this goal. In our study, couples who participated in CHTC took all other tests offered simultaneously. Completing the other tests together, learning more about each other’s broader health status and learning how to choose a healthier lifestyle e.g., improving diet and exercise, may also have improved relationships. However, we could not separately identify the effects of each individual component of health screening.

Our analysis relies on self-reported measures, which are subject to recall and social desirability biases, however, we anticipate that such biases are constant over the short study period for each couple. The generalizability of our results may be constrained because couples in our study were naïve to mutually disclosing their HIV status. Furthermore, couples who experienced any recent intimate partner violence, defined as IPV in the last six months, were not included in the IL study in recognition that they would likely need different support as part of any intervention trying to promote couples HIV testing. Further research is necessary to determine whether relationship quality is a significant predictor of CHTC participation in the general population and in relationships with a recent history of IPV. Notwithstanding these caveats and that small increases in subsequent relationship quality measures were only sustained at four months for intimacy, our analysis provides evidence of some improvement in couples’ relationship quality following CHTC and no evidence that CHTC has a negative impact on relationship quality.

## Supplementary Material

Supplementary_files

## Data Availability

The datasets generated during and/or analysed during the current study are available from the principal investigator (Nuala McGrath) on reasonable request, and also available upon request from (Alastair van Heerden).
