# Supplementary material for: Two-way associations between relationship quality and uptake of couples health screening including HIV testing and counselling together: quantitative analysis of a couples cohort in rural South Africa
Source: AIDS Care. 2024 Feb 21;36(Suppl 1):187–200. doi: 10.1080/09540121.2024.2308741 (PMC11286206; doi:10.1080/09540121.2024.2308741)
Supplement: Supplementary_files [file CAIC_A_2308741_SM9900.docx]

# Supplement material: Appendix A: Relationship quality Measures

Participants were administered a questionnaire at baseline, four weeks, and four months assessments, which included item scales to measure five relationship quality measures: satisfaction, intimacy, trust, mutual constructive communication, and conflict. The following are the item scales of each relationship quality measure. The items that were reversed during the analysis are in italic.

Satisfaction: a single-item subscale ranged from 1 (not at all satisfied) to 6 (completely satisfied).

1. In general, how satisfied are you with your relationship?

Intimacy: a six-item subscale, each ranged from 1 (Not at all true) to 9 (Very true).

1. I spend as much time with my partner as possible.
2. I do as many activities with my partner as possible.
3. I get so close to my partner that I find it hard to separate from him/her.
4. My partner is a very important part of how I see myself.
5. I think in terms of “we” and “us” instead of “I” and “me”.
6. *I can never get too close to my partner.*

Trust: an eight-item subscale, each ranged from 1 (Strongly disagree) to 7 (Strongly agree).

1. *My partner is primarily interested in his (her) own welfare.*
2. *There are times when my partner cannot be trusted.*
3. My partner is perfectly honest and truthful with me.
4. I feel that I can trust my partner completely.
5. My partner is truly sincere in his (her) promises.
6. *I feel that my partner does not show me enough consideration.*
7. My partner treats me fairly and justly.
8. I feel that my partner can be counted on to help me.

Mutual constructive communication (MCC): a three-item subscale, each ranged from 1 (Very unlikely) to 9 (Very likely).

1. When an issue or problem arises, both of us try to discuss the problem.
2. During a discussion of an issue or problem, both my partner and I express our feelings to each other.
3. During a discussion of an issue or problem, both of us suggest possible solutions and comprises.

Conflict: a one-item subscale ranged from 1 (Often) to 3 (Rarely).

1. how often do you argue?

Table 1: Participation in components of the Igugu Lethu study by enrolled couples.

| IL Study | n (%) |
| --- | --- |
| ***Marital status*** |  |
| Married | 8 (4) |
| Not married | 210 (96) |
| ***Intervention components*** | |
| **GS1** | |
| Yes | 218 (100) |
| **GS2** |  |
| Yes | 167 (76.6) |
| No | 51 (23.4) |
| **At least one couple CS** |  |
| Y­es | 113 (51.8) |
| No | 115 (48.2) |
| **Health screening, including CHTC** |  |
| Screened before four weeks assessment | 77 (35.3) |
| Screened after four weeks assessment | 45 (20.6) |
| Never screened | 96 (44.0) |
| **Post-HS CS** |  |
| Yes | 100 (81.9) |
| No | 22 (18.1) |
| **Assessment *visits*** | |
| Baseline | 218 (100) |
| Four weeks | 208 (95.4) |
| Four months | 195 (89.5) |
| **n**^£^ | 218 |

^£:^ Total number of enrolled couples in the IL study.

Table 2a: Couple-level relationship quality measures and couples’ characteristics at baseline by CHTC uptake status by four weeks and by four months.

|  | CHTC uptake by four weeks | |  |  | CHTC uptake by four months | |  |  |
| --- | --- | --- | --- | --- | --- | --- | --- | --- |
|  | Yes, by four weeks | Never / yes, after four weeks | p-Value |  | Yes | No | p-Value |  |
| **Couple-level relationship quality at baseline** | |  |  |  |  |  |  |  |
|  | Median (IQR)^a^ | |  |  | Median (IQR) ^a^ | |  |  |
| Average- satisfaction | 6 (5.5, 6) | 6 (5.5, 6) | .384 |  | 6 (5.5, 6) | 6 (5.5, 6) | .563 |  |
| Difference- satisfaction | 0 (0, 1) | 0 (0, 0) | .502 |  | 0 (0, 1) | 0 (0, 0) | .686 |  |
| Average- trust | 49.5 (45.5, 53) | 48.5 (44, 52.5) | .325 |  | 49 (45, 52.5) | 48.8 (44, 53) | .840 |  |
| Difference- trust | 3 (-3, 8) | 3 (-1, 11) | .269 |  | 4 (-2, 9) | .5 (-2.5, 11) | .512 |  |
| Average- intimacy | 48 (44.5, 52) | 47.5 (44, 52) | .227 |  | 47 (43.5, 52) | 48.3 (44.3, 52) | .416 |  |
| Difference- intimacy | 2 (0,12) | 4 (0, 12) | .565 |  | 4 (0, 14) | 4 (0, 9) | .248 |  |
| Average- communication | 25.5 (24.5, 27) | 25.5 (23, 27) | .114 |  | 25.5 (23, 27) | 25.5 (23, 27) | .802 |  |
| Difference- communication | 0 (0, 3) | 3 (-3, 8) | .256 |  | 0 (0, 3) | 0 (0, 3) | .719 |  |
| Average- conflict | 2.5 (2.5, 3) | 2.5 (2.5, 3) | .546 |  | 2.5 (2.5, 3) | 2.5 (2.5, 3) | .491 |  |
| Difference- conflict | 0 (0, 1) | 0 (0, 1) | .747 |  | 0 (0, 1) | 1 (0, 1) | .413 |  |
|  |  |  |  |  |  |  |  |  |
|  | N (%)^b^ | |  |  | N (%)^b^ | |  |  |
| **Couple age** |  |  |  |  |  |  |  |  |
| ≤ 50 years | 73 (78.5) | 116 (92.8) | .002** |  | 100 (81.9) | 89 (92.7) | .02* |  |
| 51+ years | 20 (21.5) | 9 (7.2) |  |  | 22 (18.3) | 7 (7.3) |  |  |
| **Couple education^£^** |  |  |  |  |  |  |  |  |
| Both grade 12+ | 30 (32.6) | 41 (33.1) | .349 |  | 38 (31.4) | 33 (34.7) | .374 |  |
| Both less than grade 12 | 33 (35.9) | 32 (25.8) |  |  | 42 (34.7) | 23 (24.2) |  |  |
| Male-only less than grade 12 | 11 (12.0) | 22 (17.7) |  |  | 18 (14.9) | 15 (15.8) |  |  |
| Female-only less than grad | 18 (19.6) | 29 (23.4) |  |  | 23 (19.0) | 24 (25.3) |  |  |
| **Couple employment** |  |  |  |  |  |  |  |  |
| Both employed | 7 (7.5) | 11 (8) | .931 |  | 9 (7.4) | 9 (9.4) | .887 |  |
| Neither employed | 54 (58.1) | 68 (54.4) |  |  | 69 (56.6) | 53 (55.2) |  |  |
| Male-only employed | 18 (19.4) | 24 (19.2) |  |  | 25 (20.5) | 17 (17.7) |  |  |
| Female-only employed | 14 (15.1) | 22 (17.6) |  |  | 19 (15.57) | 17 (17.7) |  |  |
| **Couple (HIV) testing history^±^** | |  |  |  |  |  |  |  |
| Both ever tested | 66 (71.7) | 93 (75) | .506 |  | 87 (71.9) | 72 (75.8) | .884 |  |
| Neither ever tested | 4 (4.3) | 9 (7.6) |  |  | 7 (5.8) | 6 (6.3) |  |  |
| Male-only tested | 6 (6.5) | 4 (3.2) |  |  | 6 (4.9) | 4 (4.2) |  |  |
| Female-only tested | 16 (17.4) | 18 (14.5) |  |  | 21 (17.4) | 13 (13.9) |  |  |
| n | 93 | 125 |  |  | 122 | 96 |  |  |

Significant level: * < .05; ** < .01. Test statistic: ^a^ Exact-Wilcoxon rank sum test; ^b^ Chi-square test. ^±^ Two couples had incomplete data on HIV testing history because one partner did not report whether they had tested for HIV in the past. ^£^ Two couples had incomplete data regarding the highest education level attained.

Table 2b: Descriptive statistics of subsequent relationship quality measures at four weeks and at four months by couples’ characteristics at baseline.

|  | Average relationship quality at four weeks | | | | | Average relationship quality at four Months | | | | |
| --- | --- | --- | --- | --- | --- | --- | --- | --- | --- | --- |
|  | Satisfaction | Trust | Intimacy | Communications | Conflict | Satisfaction | Trust | Intimacy | Communications | Conflict |
|  | Median (IQR) | | | | | Median (IQR) | | | | |
| Couples’ characteristics at baseline | | | | | |  | | | | |
| ***Couple age***^a^ |  |  |  |  |  |  |  |  |  |  |
| ≤ 50 years | 6 (5.5, 6) | 48 (44, 51.5) | 46.5 (42.5, 50) | 25.5 (23.7, 27) | 2.5 (2.5, 3) | 5.5 (5, 6) | 45.5 (41, 49.5) | 44 (40, 48) | 24.5 (22.5, 26.5) | 2.5 (2.5, 3) |
| 51+ years | 6 (5.75, 6) | 52.7 (49, 54.3) | 49.7 (47.5, 52.7) | 26.7 (25.2, 27) | 2.5 (2.2, 2.5) | 6 (5.5, 6) | 51.5 (47, 55.5) | 50 (47.5, 52) | 25 (23, 27) | 2.5 (2, 3) |
| p-value | .08 | .002** | .0002** | .08 | .52 | .01* | .0001** | .0001** | .14 | .34 |
| ***Couple education****^b,^*^£^ |  |  |  |  |  |  |  |  |  |  |
| Both grade 12+ | 6 (5.5, 6) | 46.5 (43.5, 51) | 46 (43, 49.5) | 25 (24, 27) | 2.5 (2.5, 3) | 5.5 (5, 6) | 44 (41, 49) | 44 (39, 48.5) | 24 (22, 27) | 2.5 (2.5, 3) |
| Both less than grade 12 | 6 (6, 6) | 50 (45.7, 53) | 49.5 (43, 52.5) | 26.5 (25, 27) | 2.5 (2.5, 2.5) | 5.5 (5.5, 6) | 47.5 (41.5, 52.5) | 46 (40, 50) | 25.5 (23, 27) | 2.5 (2.5, 3) |
| Male- only less than grade 12 | 6 (5.5, 6) | 48 (42.7, 52.2) | 47 (42.7, 50.5) | 25.5 (22.7, 27) | 2.5 (2.5, 3) | 5.5 (5, 6) | 46 (41.5, 48.5) | 45.2 (38, 49.5) | 24.2 (21, 25.5) | 2.5 (2.5, 3) |
| Female- only less than grade 12 | 5.5 (5.5, 6) | 48.5 (46, 51.5) | 47 (42.5, 50) | 25.5 (23, 27) | 2.5 (2.5, 2.5) | 5.5 (5, 6) | 46.5 (42, 51.5) | 46 (42.5, 47.5) | 23.5 (22.5, 26) | 2.5 (2.5, 3) |
| p-value | .05 | .05 | .054 | .34 | .71 | .60 | .27 | .46 | .02* | .39 |
| ***Couple employment*** *^b^* |  |  |  |  |  |  |  |  |  |  |
| Both employed | 6 (5.5, 6) | 47 (43.7, 52) | 46.3 (42.7, 51) | 25.5 (22.7, 27) | 2.5 (2, 2.5) | 5.5 (4.7, 6) | 46.2 (38.2, 51.7) | 44.5 (37, 49.3) | 24.2 (20.2, 27) | 2.5 (2.5, 2.5) |
| Neither employed | 6 (5.5, 6) | 48.7 (45 ,52) | 47 (43, 50) | 26 (25, 27) | 2.5 (2.5, 3) | 5.5 (5, 6) | 46.5 (42, 50.7) | 44.5 (40.2, 48) | 24.2 (22.5, 26.5) | 2.5 (2.5, 3) |
| Male-only employed | 6 (5.5, 6) | 47.5 (44.5, 51.5) | 46.5 (44, 50) | 26 (23 ,27) | 2.5 (2, 2.5) | 5.5 (5, 6) | 43.5 (39.5, 50) | 46 (39, 50) | 25 (22.5, 26.5) | 2.5 (2.5, 3) |
| Female-only employed | 6 (5.5, 6) | 51 (45, 53) | 49.5 (44.5, 51) | 25.5 (23.5, 26.5) | 2.5 (2.5, 3) | 5.5 (5.5, 6) | 47 (39.5, 52.5) | 44 (41.5, 49.5) | 24.5 (22.5, 27) | 2.5 (2.5, 2.5) |
| p-value | .47 | .57 | .88 | .47 | .12 | .36 | .68 | .90 | .95 | .07 |
| ***Couple (HIV) testing*** *history^b,^*^£^ | | |  |  |  |  |  |  |  |  |
| Both ever tested | 6 (5.5, 6) | 45 (44, 52) | 46.5 (42.5, 50) | 25.5 (23, 27) | 2.5 (2.3, 3) | 5.5 (5, 6) | 44.5 (41, 50.3) | 44 (39.5, 48) | 24.3 (22, 27) | 2.5 (2.5, 3) |
| Neither ever tested | 6 (5.5, 6) | 52 (46.5, 53) | 51.5 (40.5, 52.5) | 27 (27, 27) | 2.5 (2.5, 2.5) | 6 (5.5, 6) | 47.5 (39.5, 53) | 44.5 (40, 52) | 23 (23, 25) | 2.5 (2.5, 3) |
| Male-only tested | 6 (6, 6) | 49.5 (47, 51.5) | 47 (45, 52) | 26 (25.5, 27) | 2(2, 2.5) | 6 (5.5, 6) | 48.5 (42, 50) | 46.5 (44, 50) | 25 (22.5, 26.5) | 2.5 (2.5, 3) |
| Female-only tested | 6 (5.5, 6) | 49.5 (45.5, 52) | 47 (45.5, 50) | 25.5 (23.5, 27) | 2.5 (2.5, 3) | 5.5 (5, 6) | 47.5 (45.7, 41) | 46 (43.3, 48.5) | 25.3 (23, 27) | 2.5 (2.5, 3) |
| P-value | 0.40 | 0.19 | 0.49 | 0.04* | 0.21 | 0.48 | 0.27 | 0.28 | 0.30 | 0.66 |
| N | 208^a^ | 208^a^ | 208^a^ | 208^a^ | 208^a^ | 195^b^ | 195^b^ | 195^b^ | 195^b^ | 195^b^ |

Significant level: * < .05; ** < .01.

test statistic: ^a^ Exact-Wilcoxon rank sum test; ^b^ Kruskal-Wallis test.

^±^ Two couples had incomplete data on HIV testing history because one partner did not report whether they had tested for HIV in the past.

^£^ Two couples had incomplete data regarding the highest education level attained.

^a^ Total number of couples who completed 4 weeks assessment; ^b^ Total number of couples who completed 4 months assessmentز

Table 3a: Final Multivariate logistic regressions examining the association between relationship quality measures at baseline and the odds of CHTC uptake by four weeks.

|  | (1) | (2) | (3) | (4) | (5) |
| --- | --- | --- | --- | --- | --- |
|  | CHTC-4w  OR (95% CI) | CHTC-4w  OR (95% CI) | CHTC-4w  OR (95% CI) | CHTC-4w  OR (95% CI) | CHTC-4w  OR (95% CI) |
| ***Couple-level relationship quality at baseline*** |  |  |  |  |  |
| Average- satisfaction | 1.88 |  |  |  |  |
|  | (0.89 - 4.01) |  |  |  |  |
| Difference- satisfaction | 1.27 |  |  |  |  |
|  | (0.84 - 1.92) |  |  |  |  |
| Average- trust |  | 1.00 |  |  |  |
|  |  | (0.95 - 1.06) |  |  |  |
| Difference- trust |  | 0.98 |  |  |  |
|  |  | (0.95 - 1.01) |  |  |  |
| Average- intimacy |  |  | 1.03 |  |  |
|  |  |  | (0.97 - 1.10) |  |  |
| Difference- intimacy |  |  | 1.00 |  |  |
|  |  |  | (0.97 - 1.04) |  |  |
| Average- communication |  |  |  | 1.06 |  |
|  |  |  |  | (0.95 - 1.19) |  |
| Difference- communication |  |  |  | 0.97 |  |
|  |  |  |  | (0.91 - 1.03) |  |
| Average- conflict |  |  |  |  | 0.70 |
|  |  |  |  |  | (0.31 - 1.61) |
| Difference- conflict |  |  |  |  | 0.87 |
|  |  |  |  |  | (0.59 - 1.30) |
| ***Couple age*** |  |  |  |  |  |
| ≤ 50 years | reference | reference | reference | reference | reference |
| 51+ years | 3.64** | 3.81** | 3.54** | 3.54** | 3.66** |
|  | (1.44 - 9.19) | (1.48 - 9.78) | (1.40 - 8.95) | (1.40 - 8.96) | (1.46 - 9.21) |
| ***Couple education*** |  |  |  |  |  |
| Both grade 12+ | reference | reference | reference | reference | reference |
| Both less than grade 12 | 0.92 | 1.01 | 0.97 | 1.00 | 1.02 |
|  | (0.43 - 1.97) | (0.47 - 2.17) | (0.45 - 2.09) | (0.47 - 2.15) | (0.48 - 2.19) |
| Male- only less than grade 12 | 0.68 | 0.71 | 0.70 | 0.74 | 0.72 |
|  | (0.27 - 1.69) | (0.29 - 1.76) | (0.28 - 1.73) | (0.30 - 1.84) | (0.29 - 1.78) |
| Female-only less than grade 12 | 0.74 | 0.79 | 0.76 | 0.73 | 0.81 |
|  | (0.33 - 1.65) | (0.35 - 1.77) | (0.34 - 1.68) | (0.33 - 1.62) | (0.37 - 1.81) |
| ***Couple employment*** |  |  |  |  |  |
| Both employed | 0.70 | 0.69 | 0.68 | 0.73 | 0.60 |
|  | (0.24 - 2.08) | (0.23 - 2.07) | (0.23 - 1.98) | (0.24 - 2.17) | (0.19 - 1.86) |
| Neither employed | reference | reference | reference | reference | reference |
| Male-only employed | 0.97 | 1.01 | 0.99 | 1.09 | 1.01 |
|  | (0.46 - 2.04) | (0.48 - 2.12) | (0.47 - 2.08) | (0.51 - 2.32) | (0.48 - 2.12) |
| Female-only employed | 0.80 | 0.74 | 0.77 | 0.77 | 0.77 |
|  | (0.35 - 1.83) | (0.33 - 1.66) | (0.34 - 1.76) | (0.34 - 1.72) | (0.34 - 1.74) |
| ***Couple (HIV) testing history*** |  |  |  |  |  |
| Both never tested | reference | reference | reference | reference | reference |
| Neither ever tested | 0.48 | 0.50 | 0.51 | 0.53 | 0.52 |
|  | (0.13 - 1.76) | (0.14 - 1.85) | (0.14 - 1.88) | (0.14 - 2.00) | (0.14 - 1.95) |
| Male-only tested | 2.29 | 1.95 | 2.04 | 1.98 | 2.11 |
|  | (0.56 - 9.40) | (0.50 - 7.59) | (0.53 - 7.91) | (0.52 - 7.56) | (0.55 - 8.09) |
| Female-only tested | 1.25 | 1.30 | 1.39 | 1.35 | 1.35 |
|  | (0.56 - 2.80) | (0.58 - 2.90) | (0.62 - 3.11) | (0.60 - 3.04) | (0.60 - 3.02) |
| n^£^ | 213 | 213 | 213 | 213 | 213 |

Significant level: * < .05; ** < .01.

^£^ The models exclude: two couple had incomplete data on HIV testing history because one partner did not report whether they had tested for HIV in the past, another couple was excluded where the female partner reported a disclosure of her positive HIV test result to her partner, and another Two couples had incomplete data re: education level attained.

Table 3b: Final Multivariate logistic regressions examining the association between relationship quality measures at baseline and the odds of CHTC uptake by four months.

|  | (1) | | (2) | | (3) | | (4) | | (5) | |
| --- | --- | --- | --- | --- | --- | --- | --- | --- | --- | --- |
|  | CHTC-4m  OR (95% CI) | | CHTC-4m  OR (95% CI) | | CHTC-4m  OR (95% CI) | | CHTC-4m  OR (95% CI) | | CHTC-4m  OR (95% CI) | |
| ***Couple-level relationship quality at baseline*** | | |  | |  | |  | |  | |
| Average- satisfaction | | 1.35 | |  | |  | |  | |  |
|  | | (0.76 - 2.38) | |  | |  | |  | |  |
| Difference- satisfaction | | 1.05 | |  | |  | |  | |  |
|  | | (0.77 - 1.43) | |  | |  | |  | |  |
| Average- trust | |  | | 1.00 | |  | |  | |  |
|  | |  | | (0.95 - 1.05) | |  | |  | |  |
| Difference- trust | |  | | 1.00 | |  | |  | |  |
|  | |  | | (0.98 - 1.03) | |  | |  | |  |
| Average- intimacy | |  | |  | | 0.99 | |  | |  |
|  | |  | |  | | (0.93 - 1.05) | |  | |  |
| Difference- intimacy | |  | |  | | 1.02 | |  | |  |
|  | |  | |  | | (0.99 - 1.06) | |  | |  |
| Average- communication | |  | |  | |  | | 0.98 | |  |
|  | |  | |  | |  | | (0.88 - 1.09) | |  |
| Difference- communication | |  | |  | |  | | 0.98 | |  |
|  | |  | |  | |  | | (0.92 - 1.04) | |  |
| Average- conflict | |  | |  | |  | |  | | 0.63 |
|  | |  | |  | |  | |  | | (0.27 - 1.46) |
| Difference- conflict | |  | |  | |  | |  | | 0.79 |
|  | |  | |  | |  | |  | | (0.52 - 1.19) |
| ***Couple age*** | |  | |  | |  | |  | |  |
| ≤ 50 years | | reference | | reference | | reference | | reference | | reference |
| 51+ years | 2.63 | | 2.68* | | 2.84* | | 2.77* | | 2.65* | |
|  | (1.00 - 6.93) | | (1.00 - 7.14) | | (1.07 - 7.51) | | (1.05 - 7.31) | | (1.01 - 6.98) | |
| ***Couple Education*** |  | |  | |  | |  | |  | |
| Both grade 12+ | reference | | reference | | reference | | reference | | reference | |
| Both less than grade 12 | 1.15 | | 1.21 | | 1.24 | | 1.22 | | 1.23 | |
|  | (0.54 - 2.45) | | (0.57 - 2.57) | | (0.58 - 2.66) | | (0.58 - 2.59) | | (0.58 - 2.61) | |
| Male-only less than grade 12 | 0.98 | | 0.99 | | 0.91 | | 0.99 | | 1.05 | |
|  | (0.41 - 2.32) | | (0.42 - 2.34) | | (0.38 - 2.18) | | (0.42 - 2.36) | | (0.44 - 2.50) | |
| Female-only less than grade 12 | 0.76 | | 0.75 | | 0.78 | | 0.75 | | 0.83 | |
|  | (0.35 - 1.63) | | (0.35 - 1.63) | | (0.36 - 1.68) | | (0.35 - 1.62) | | (0.38 - 1.80) | |
| ***Couple employment*** |  | |  | |  | |  | |  | |
| Both employed | 0.68 | | 0.70 | | 0.75 | | 0.67 | | 0.56 | |
|  | (0.24 - 1.94) | | (0.25 - 1.99) | | (0.26 - 2.15) | | (0.24 - 1.91) | | (0.19 - 1.68) | |
| Neither employed | reference | | reference | | reference | | reference | | reference | |
| Male-only employed | 1.15 | | 1.18 | | 1.23 | | 1.22 | | 1.17 | |
|  | (0.55 - 2.39) | | (0.57 - 2.45) | | (0.59 - 2.56) | | (0.58 - 2.55) | | (0.56 - 2.44) | |
| Female-only employed | 0.81 | | 0.84 | | 0.92 | | 0.82 | | 0.80 | |
|  | (0.37 - 1.80) | | (0.38 - 1.84) | | (0.41 - 2.02) | | (0.37 - 1.78) | | (0.36 - 1.75) | |
| ***Couple (HIV) testing history*** |  | |  | |  | |  | |  | |
| Both ever tested | reference | | reference | | reference | | reference | | reference | |
| Neither ever tested | 0.75 | | 0.81 | | 0.78 | | 0.84 | | 0.83 | |
|  | (0.23 - 2.46) | | (0.24 - 2.66) | | (0.24 - 2.59) | | (0.25 - 2.76) | | (0.25 - 2.76) | |
| Male-only tested | 1.16 | | 1.15 | | 1.21 | | 1.11 | | 1.20 | |
|  | (0.30 - 4.54) | | (0.30 - 4.41) | | (0.32 - 4.64) | | (0.29 - 4.26) | | (0.31 - 4.61) | |
| Female-only tested | 1.34 | | 1.39 | | 1.34 | | 1.39 | | 1.40 | |
|  | (0.59 - 3.03) | | (0.62 - 3.12) | | (0.59 - 3.03) | | (0.62 - 3.13) | | (0.62 - 3.15) | |
| n^£^ | 213 | | 213 | | 213 | | 213 | | 213 | |

Significant level: * < .05; ** < .01.

^£^ The models exclude: two couple had incomplete data on HIV testing history because one partner did not report whether they had tested for HIV in the past, another couple was excluded where the female partner reported a disclosure of her positive HIV test result to her partner, and another Two couples had incomplete data re: education level attained.

Table 4a: Final Multivariate linear regressions examining the association between the intervention components, CHTC uptake and each relationship quality measure at four weeks.

|  | (1) | (2) | (3) | (4) | (5) |
| --- | --- | --- | --- | --- | --- |
|  | Satisfaction  β (95% CI) | Trust  β (95% CI) | Intimacy  β (95% CI) | Communication  β (95% CI) | Conflict  β (95% CI) |
| ***Intervention components*** | |  |  |  |  |
| ***GS2*** |  |  |  |  |  |
| No | reference | Reference | reference | reference | reference |
| Yes | 0.05 | -0.05 | 0.36 | 0.35 | 0.08 |
|  | (-0.11 - 0.21) | (-1.68 - 1.58) | (-1.33 - 2.05) | (-0.56 - 1.27) | (-0.05 - 0.20) |
| ***At least one couple CS before 4 weeks visit*** | |  |  |  |  |
| No | reference | Reference | reference | reference | reference |
| Yes | 0.17* | 0.84 | 1.97* | 0.22 | -0.22** |
|  | (0.00 - 0.33) | (-0.82 - 2.50) | (0.22 - 3.72) | (-0.71 - 1.16) | (-0.35 - -0.10) |
| ***Health Screening, including CHTC*** | |  |  |  |  |
| Never/Screened after 4 weeks visit | reference | Reference | reference | reference | reference |
| Screened before 4 weeks visit | 0.21** | 1.99** | 1.51 | 0.60 | 0.01 |
|  | (0.07 - 0.36) | (0.55 - 3.44) | (-0.00 - 3.03) | (-0.22 - 1.42) | (-0.10 - 0.12) |
| ***Couple (HIV) testing history^±^*** | |  |  |  |  |
| Both ever tested | reference | Reference | reference | reference | reference |
| Neither ever tested | 0.06 | 2.56 | 0.58 | 1.63* | 0.07 |
|  | (-0.21 - 0.34) | (-0.20 - 5.33) | (-2.30 - 3.45) | (0.06 - 3.21) | (-0.14 - 0.28) |
| Male-only tested | 0.20 | 0.76 | 0.77 | 1.18 | -0.35** |
|  | (-0.14 - 0.53) | (-2.55 - 4.06) | (-2.66 - 4.20) | (-0.69 - 3.04) | (-0.60 - -0.10) |
| Female-only tested | 0.11 | 2.37* | 3.05** | -0.06 | 0.00 |
|  | (-0.08 - 0.30) | (0.46 - 4.27) | (1.07 - 5.03) | (-1.13 - 1.01) | (-0.14 - 0.14) |
| **Couple-level relationship quality at baseline** | | | | | |
| Average- satisfaction | 0.25** |  |  |  |  |
|  | (0.12 - 0.38) |  |  |  |  |
| Difference- satisfaction | 0.03 |  |  |  |  |
|  | (-0.05 - 0.10) |  |  |  |  |
| Average- trust |  | 0.50** |  |  |  |
|  |  | (0.39 - 0.62) |  |  |  |
| Difference- trust |  | 0.01 |  |  |  |
|  |  | (-0.05 - 0.08) |  |  |  |
| Average- intimacy |  |  | 0.50** |  |  |
|  |  |  | (0.35 - 0.64) |  |  |
| Difference- intimacy |  |  | -0.01 |  |  |
|  |  |  | (-0.09 - 0.08) |  |  |
| Average- communication |  |  |  | 0.32** |  |
|  |  |  |  | (0.18 - 0.46) |  |
| Difference- communication |  |  |  | 0.03 |  |
|  |  |  |  | (-0.04 - 0.11) |  |
| Average- conflict |  |  |  |  | 0.18* |
|  |  |  |  |  | (0.03 - 0.33) |
| Difference- conflict |  |  |  |  | -0.02 |
|  |  |  |  |  | (-0.09 - 0.05) |
| n^£^ | 203 | 203 | 203 | 203 | 203 |

Significant level: * < .05; ** < .01.

^£^ 203 couples had completed relationship quality questions at baseline and four weeks assessments. The models exclude: two couple had incomplete data on HIV testing history because one partner did not report whether they had tested for HIV in the past, another couple was excluded where the female partner reported a disclosure of her positive HIV test result to her partner, and another two couples had incomplete regarding the highest education level attained.

Table 4b: Final Multivariate linear regressions examining the association between the intervention components, CHTC uptake and each relationship quality measure at four Months.

|  | (1) | (2) | (3) | (4) | (5) |
| --- | --- | --- | --- | --- | --- |
|  | Satisfaction  β (95% CI) | Trust  β (95% CI) | Intimacy  β (95% CI) | Communication  β (95% CI) | Conflict  β (95% CI) |
| ***Intervention components*** |  |  |  |  |  |
| **GS2** |  |  |  |  |  |
| No | reference | reference | reference | reference | reference |
| Yes | -0.14 | 0.35 | -0.79 | -0.64 | 0.10 |
|  | (-0.38 - 0.10) | (-1.78 - 2.48) | (-2.70 - 1.13) | (-1.63 - 0.35) | (-0.02 - 0.22) |
| ***At least one couple CS*** |  |  |  |  |  |
| No | reference | reference | reference | reference | reference |
| Yes | 0.19 | 1.14 | 2.20* | -0.22 | 0.06 |
|  | (-0.05 - 0.42) | (-0.94 - 3.21) | (0.35 - 4.04) | (-1.18 - 0.75) | (-0.06 - 0.18) |
|  |  |  |  |  |  |
| ***Health Screening, including CHTC*** | |  |  |  |  |
| Never | reference | reference | reference | reference | reference |
| Screened after 4 weeks visit | 0.08 | 0.86 | 4.24** | -0.09 | 0.03 |
|  | (-0.27 - 0.43) | (-2.28 - 4.01) | (1.42 - 7.07) | (-1.56 - 1.38) | (-0.15 - 0.21) |
| Screened before 4 weeks visit | 0.14 | 1.00 | 4.15* | -0.16 | -0.13 |
|  | (-0.28 - 0.55) | (-2.67 - 4.67) | (0.88 - 7.43) | (-1.87 - 1.54) | (-0.34 - 0.08) |
| ***Post-HS CS*** |  |  |  |  |  |
| No | reference | reference | reference | reference | reference |
| Yes | 0.17 | 2.20 | 0.09 | 0.71 | 0.13 |
|  | (-0.18 - 0.52) | (-0.90 - 5.30) | (-2.67 - 2.84) | (-0.72 - 2.15) | (-0.05 - 0.30) |
| ***Couples age*** |  |  |  |  |  |
| ≤ 50 years | reference | reference | reference | reference | reference |
| 51+ years | 0.19 | 4.06** | 4.20** | 0.54 | -0.06 |
|  | (-0.12 - 0.49) | (1.27 - 6.84) | (1.80 - 6.61) | (-0.71 - 1.80) | (-0.21 - 0.10) |
| ***Couples (HIV) testing history*** | |  |  |  |  |
| Both tested | reference | reference | reference | reference | reference |
| Neither tested | 0.13 | 0.71 | 0.37 | -0.36 | 0.07 |
|  | (-0.27 - 0.53) | (-2.84 - 4.27) | (-2.77 - 3.51) | (-2.01 - 1.29) | (-0.13 - 0.27) |
| Male-only tested | 0.19 | 1.07 | 1.93 | 0.63 | 0.13 |
|  | (-0.29 - 0.67) | (-3.15 - 5.29) | (-1.82 - 5.68) | (-1.33 - 2.59) | (-0.11 - 0.37) |
| Female-only tested | 0.21 | 2.65* | 2.87* | 1.07 | -0.06 |
|  | (-0.07 - 0.49) | (0.18 - 5.13) | (0.68 - 5.07) | (-0.08 - 2.21) | (-0.20 - 0.08) |
| **Couple-level relationship Quality at baseline** | |  |  |  |  |
| Average- satisfaction | 0.11 |  |  |  |  |
|  | (-0.11 - 0.33) |  |  |  |  |
| Difference- satisfaction | -0.17** |  |  |  |  |
|  | (-0.28 - -0.05) |  |  |  |  |
| Average- trust |  | 0.22* |  |  |  |
|  |  | (0.05 - 0.39) |  |  |  |
| Difference- trust |  | -0.02 |  |  |  |
|  |  | (-0.12 - 0.07) |  |  |  |
| Average- intimacy |  |  | 0.24** |  |  |
|  |  |  | (0.07 - 0.41) |  |  |
| Difference- intimacy |  |  | -0.09 |  |  |
|  |  |  | (-0.19 - 0.01) |  |  |
| Average- communication |  |  |  | 0.10 |  |
|  |  |  |  | (-0.06 - 0.26) |  |
| Difference- communication |  |  |  | -0.05 |  |
|  |  |  |  | (-0.13 - 0.04) |  |
| Average- conflict |  |  |  |  | 0.11 |
|  |  |  |  |  | (-0.04 - 0.26) |
| Difference- conflict |  |  |  |  | 0.03 |
|  |  |  |  |  | (-0.04 - 0.10) |
| n^£^ | 191 | 191 | 191 | 191 | 191 |

Significant level: * < .05; ** < .01.

^£^ 191 couples had completed relationship quality questions at baseline and four months assessments. The models exclude: two couple had incomplete data on HIV testing history because one partner did not report whether they had tested for HIV in the past, another couple was excluded where the female partner reported a disclosure of her positive HIV test result to her partner, and another Two couples had incomplete data regarding the highest education level attained.

Table 5a: Final Multivariate linear regressions examining the association between the intervention components, and HIV result from CHTC uptake before four weeks and each relationship quality measure at four weeks^#^.

|  | (1) | (2) | (3) | (4) | (5) |
| --- | --- | --- | --- | --- | --- |
|  | Satisfaction  β (95% CI) | Trust  β (95% CI) | Intimacy  β (95% CI) | Communication  β (95% CI) | Conflict  β (95% CI) |
|  |  |  |  |  |  |
|  |  |  |  |  |  |
| ***Health Screening, including CHTC*** |  |  |  |  |  |
| Never/Screened after 4 weeks visit | reference | reference | reference | reference | Reference |
| Two HIV-negative results at CHTC (n=26) | 0.23* | 1.00 | 1.16 | 0.68 | 0.10 |
|  | (0.02 - 0.44) | (-1.06 - 3.06) | (-1.01 - 3.32) | (-0.49 - 1.84) | (-0.06 - 0.26) |
| One HIV-positive result at CHTC (n=22) | 0.23* | 3.22** | 1.85 | 1.07 | -0.05 |
|  | (0.01 - 0.45) | (1.02 - 5.41) | (-0.46 - 4.16) | (-0.17 - 2.31) | (-0.21 - 0.12) |
| Two HIV-positive results at CHTC (n=18) | 0.16 | 1.90 | 1.62 | -0.08 | -0.03 |
|  | (-0.08 - 0.40) | (-0.47 - 4.28) | (-0.89 - 4.12) | (-1.44 - 1.28) | (-0.21 - 0.15) |
| N^£^ | 203 | 203 | 203 | 203 | 203 |

^#^ The results are from models also adjusted for the intervention components, couples HIV testing history, and the corresponding couple-level relationship quality measure at baseline, data not shown.

Significant level: * < .05; ** < .01.

^£^ 203 couples had completed relationship quality questions at baseline and four weeks assessments. The models exclude: two couple had incomplete data on HIV testing history because one partner did not report whether they had tested for HIV in the past, another couple was excluded where the female partner reported a disclosure of her positive HIV test result to her partner, and another two couples had incomplete regarding the highest education level attained.

Table 5b: Final Multivariate linear regressions examining the association between the intervention components, HIV result from CHTC uptake at any time during the follow up and each relationship quality measure at four Months^#^.

|  | (1) | (2) | (3) | (4) | (5) |
| --- | --- | --- | --- | --- | --- |
|  | Satisfaction  β (95% CI) | Trust  β (95% CI) | Intimacy  β (95% CI) | Communication  β (95% CI) | Conflict  β (95% CI) |
|  |  |  |  |  |  |
|  |  |  |  |  |  |
| ***Health Screening, including CHTC*** |  |  |  |  |  |
| Never | reference | Reference | reference | reference | reference |
| Two HIV-negative results at CHTC (n=44) | 0.08 | -0.24 | 3.10* | -0.60 | -0.03 |
|  | (-0.30 - 0.46) | (-3.61 - 3.13) | (0.11 - 6.10) | (-2.17 - 0.97) | (-0.23 - 0.16) |
| One HIV-positive result at CHTC (n=39) | 0.07 | 1.45 | 5.03** | 0.58 | -0.02 |
|  | (-0.32 - 0.47) | (-2.06 - 4.96) | (1.89 - 8.17) | (-1.05 - 2.20) | (-0.22 - 0.19) |
| Two HIV-positive results at CHTC (n=31) | 0.13 | 1.91 | 5.03** | -0.11 | 0.03 |
|  | (-0.27 - 0.53) | (-1.65 - 5.46) | (1.86 - 8.20) | (-1.75 - 1.54) | (-0.18 - 0.24) |
| N^£^ | 191 | 191 | 191 | 191 | 191 |

^#^ The results are from models also adjusted for the intervention components, post-HS CS, couple age, couple HIV testing history, data not shown.

Significant level: * < .05; ** < .01.

^£^ 191 couples had completed relationship quality questions at baseline and four months assessments. The models exclude: two couple had incomplete data on HIV testing history because one partner did not report whether they had tested for HIV in the past, another couple was excluded where the female partner reported a disclosure of her positive HIV test result to her partner, and another Two couples had incomplete data regarding the highest education level attained.
